# Genome-partitioning strategy, plastid and nuclear phylogenomic discordance, and its evolutionary implications of *Clematis* (Ranunculaceae)

**DOI:** 10.3389/fpls.2022.1059379

**Published:** 2022-11-14

**Authors:** Jiamin Xiao, Rudan Lyu, Jian He, Mingyang Li, Jiaxin Ji, Jin Cheng, Lei Xie

**Affiliations:** ^1^ School of Ecology and Nature Conservation, Beijing Forestry University, Beijing, China; ^2^ College of Biological Sciences and Technology, Beijing Forestry University, Beijing, China

**Keywords:** *Clematis*, cyto-nuclear discordance, genome partitioning, genome skimming, transcriptome, phylogenomics

## Abstract

*Clematis* is one of the largest genera of Ranunculaceae with many phylogenetic problems left to be resolved. *Clematis* species have considerable genome size of more than 7 Gbp, and there was no whole-genome reference sequence published in this genus. This raises difficulties in acquiring nuclear genome data for its phylogenetic analysis. Previous studies based on Sanger sequencing data, plastid genome data, and nrDNA sequences did not well resolve the phylogeny of *Clematis*. In this study, we used genome skimming and transcriptome data to assemble the plastid genome sequences, nuclear single nucleotide polymorphisms (SNPs) datasets, and single-copy nuclear orthologous genes (SCOGs) to reconstruct the phylogenetic backbone of *Clematis*, and test effectiveness of these genome partitioning methods. We also further analyzed the discordance among nuclear gene trees and between plastid and nuclear phylogenies. The results showed that the SCOGs datasets, assembled from transcriptome method, well resolved the phylogenetic backbone of *Clematis*. The nuclear SNPs datasets from genome skimming method can also produce similar results with the SCOGs data. In contrast to the plastid phylogeny, the phylogeny resolved by nuclear genome data is more robust and better corresponds to morphological characters. Our results suggested that rapid species radiation may have generated high level of incomplete lineage sorting, which was the major cause of nuclear gene discordance. Our simulation also showed that there may have been frequent interspecific hybridization events, which led to some of the cyto-nuclear discordances in *Clematis*. This study not only provides the first robust phylogenetic backbone of *Clematis* based on nuclear genome data, but also provides suggestions of genome partitioning strategies for the phylogenomic study of other plant taxa.

## Introduction

With the rapid development of molecular biotechnology, the cost of high-throughput sequencing continues to decrease. Using genomic data to reconstruct phylogeny and explore the origin and evolutionary history of plant taxa is growing rapidly (Zimmer and Wen, 2015; Wen et al., 2017; Marks et al., 2021; Kress et al., 2022). Compared to previous studies using the Sanger sequencing method, the application of genomic data has greatly improved the resolution of the phylogenetic trees (Valcárcel and Wen, 2019; Li et al., 2021; Khan et al., 2021). Genomic data can not only generate better resolved phylogenies of plant taxa, but can also alleviate the problem of stochastic error due to insufficient information from small datasets (Yu et al., 2018; Watson et al., 2020).

In recent years, the plastid genome (plastome) has been considered to be the most important source of data and widely applied for phylogenetic reconstruction of green plant phylogeny at almost all taxonomic levels (Li et al., 2019; Zhai et al., 2019; Zhang et al., 2020; Zhao et al., 2021). However, the uni-parental inherited plastid genome sometimes showed conflicting phylogenetic signals with the bi-parental inherited nuclear genome data (cyto-nuclear discordance) due to chloroplast capture, incomplete lineage sorting (ILS) (Rose et al., 2021), or other factors such as sampling error, stochastic error, paralogs, and so on (Zou and Ge, 2008). Comprehensive understanding of evolutionary process for a plant taxon requires both cytoplasmic and nuclear genome evidence and an in depth analysis of their phylogenetic discordance (Lee-Yaw et al., 2019).

*Clematis* L. is one of the largest genera in the family Ranunculaceae with about 300 wild species, most of which are diploid (Tamura, 1995; Wang and Bartholomew, 2001; Wang and Li, 2005). The taxonomy of *Clematis* has been considered to be difficult. Many classifications published in recent years held different views on many issues, including the delineation of the genus, infrageneric classification, and species delimitation (Tamura, 1995; Johnson, 1997; Grey-Wilson, 2000; Wang and Li, 2005). Previous molecular phylogenetic studies, based on the nuclear ribosomal DNA (nrDNA), the plastid fragments, and the complete plastome data, have solved many of those problems, such as genus delineation and the identification of the sister group of *Clematis* (Miikeda et al., 2006; Xie et al., 2011; Lehtonen et al., 2016; Jiang et al., 2017; He et al., 2021). However, all the previously published studies had limitations of not establishing a robust phylogenetic framework within *Clematis*, and its extensive cyto-nuclear discordance remains to be analyzed by inclusion of more nuclear genome data.

There are several reasons that may contribute to the difficulties in reconstructing a robust phylogeny of *Clematis*. Firstly, according to previous molecular studies, species radiation events may have happened during the late Neogene and the Quaternary (Xie et al., 2011; He et al., 2021). Small number of DNA sequences with insufficient informative loci often failed to resolve the relationships among recently radiated groups (Zhao et al., 2021). Secondly, interspecific hybridization may have happened or may be not uncommon in *Clematis* (Lyu et al., 2021), that may cause cyto-nuclear discordance during phylogenetic reconstruction. Thirdly, *Clematis* species have relatively large genome size (7.18 Gbp−16.43 Gbp, https://cvalues.science.kew.org/search) and there is no high-quality whole genome data available, which raise technical difficulties for genome-partitioning selection.

The genome-partitioning methods for phylogenomic study of plant taxa generally include reduced-representation Genome Sequencing (RRGS), genome skimming, transcriptome sequencing or RNA-seq, and target enrichment sequencing (Zimmer and Wen, 2015; Yu et al., 2018; McKain et al., 2018). Among them, genome skimming, which randomly captures a certain percentage of total genomic DNA (Dodsworth, 2015; Thode et al., 2020; Wikström et al., 2020), has been widely applied for phylogenetic studies (Wen et al., 2018; Su et al., 2021; He et al., 2021). One of the advantages of genome skimming method is that fresh, silica-gel dried, or even herbarium materials can be used for this method (Liu et al., 2019; Wang et al., 2020). Using genome skimming data, cytoplasmic genome and tandemly repeated nrDNA can be assembled for phylogenetic reconstruction (Yu et al., 2018; Fonseca and Lohmann, 2020). According to the recently developed method by Liu et al. (2021), genome skimming data with high sequencing depth (10 × or more) can be used for assembling single-copy nuclear genes for phylogenetic studies. Other studies have shown that genome skimming data with low sequencing depth (less than 1 ×) can be used to obtain single nucleotide polymorphisms (SNPs) from nuclear genome for phylogenetic reconstruction (Olofsson et al., 2019).

In contrast, transcriptome method has irreplaceable advantages for obtaining single-copy nuclear genes (One Thousand Plant Transcriptomes Initiative, 2019), and plant genome size is not the factor affecting sequencing depth because the transcribed gene content is small and very stable among seed plants (around 0.03 Gbp, Novák et al., 2020). However, the application of the transcriptome method is limited by plant material, which requires fresh plant tissue (or stored in RNA stabilization solution), or at least silica gel dried material (He et al., 2022). For a large genus like *Clematis*, a considerable proportion of species samples may be from herbarium specimens. It is difficult to obtain transcriptome data from all samples. In recent years, using targeted enrichment sequencing method to obtain nuclear gene has attracted much attention in phylogenetic studies (Vargas et al., 2019; Stull et al., 2020). This method can also use herbarium material for DNA extraction. However, comparing to RNA-seq method, the target enrichment method has much more complicated experimental process, relatively smaller amount of data, too much missing data, and low data reusability (McKain et al., 2018).

For *Clematis*, an accurate and well-supported phylogenetic backbone still remains to be reconstructed by nuclear genome data. Obtaining high-depth sequencing data (10 × means at least 70 Gbp for each sample in *Clematis*) to assemble nuclear genes is not economically viable for *Clematis*. In this study, using genome skimming (with low depth) and transcriptome data, we try to answer the following questions: to what extent nuclear genome data may improve the phylogenetic inference of *Clematis*? can genome skimming data with low sequencing depth provide more nuclear phylogenetic information? if the nuclear single nucleotide polymorphisms (SNPs) data from genome skimming method can be used for *Clematis* phylogenetic reconstruction? which one, incomplete lineage sorting or hybridization, may have caused the cyto-nuclear discordance of *Clematis*? This study will also shed light on the genome partitioning selection for phylogenomic analysis of other similar taxa with recent species radiation and considerable genome size.

## Materials and methods

### Plant material

Because the major purpose of this study is to check the robustness of the phylogenetic backbones inferred by different datasets, we chose a phylogenetically representative sampling scheme with only key species of *Clematis* in this study. A total of 32 species (about 1/10 of total species) were used for our phylogenomic analysis, covering all the subgenera of both Tamura (1995) and Wang and Li (2005). This sampling scheme also covers 11 sections (of the total 17) in the classification of Tamura (1995), and 9 sections (of the total 15) in the classification of Wang and Li (2005). Although we did not include several small sections (like sect. *Archiclematis*, sect. *Pterocarpa*, and sect. *Angustifoliae*), our sampling represented all the major lineages (clades) of *Clematis* included in previous studies (Miikeda et al., 2006; Xie et al., 2011; He et al., 2021). Furthermore, our previous studies showed that some sections, such as sect. *Clematis* and sect. *Viorna* (Reichb.) Prantl (sensu Wang and Li, 2005), may be polyphyletic. So, our samples also included problematic species of those sections (**Supplementary Table S1**).

The plant materials are mostly collected from the field, only with two samples from herbarium specimens. Among all the 32 sampled species, genome skimming data of 28 were newly generated for this study, and those of the other four species were retrieved from previous studies (**Supplementary Table S1**). Because specimen materials cannot yield RNA-seq data, transcriptomes of only 28 species were sequenced in this study. According to Jiang et al. (2017), *Anemoclema glaucifolium* (Franch.) W. T. Wang was chosen as an outgroup.

### Methods for genomic data acquisition

#### Transcriptome sequencing

Transcriptome sequencing followed the method of He et al. (2022). Total RNAs were extracted at Biomarker Technologies Corporation (https://www.biomarker.com.cn) from silica gel dried leaves using TRIzon Reagent (TRIzon, CoWin Biosciences, Jiangsu, PR China). Then the RNAs were reversed into cDNA, and paired-end reads of 2 ×150 libraries were generated and sequenced on a NovaSeq 6000 platform (Illumina, San Diego, California, USA). About 6 Gbp of raw reads were obtained for each samples. The raw reads were then filtered and trimmed using fastp v.0.20 (Chen et al., 2018). The clean transcriptomes were *de novo* assembled using Trinity v.2.5.1 (Grabherr et al., 2011) with default parameters. All the transcriptome data were deposited in GenBank (**Supplementary Table S2**).

#### Genome skimming sequencing

The total genomic DNAs were extracted from silica-dried samples at Biomarker Technologies Corporation (https://www.biomarker.com.cn) using a genomic DNA extraction kit following manufacturer instructions (Tiangen Biotech Co. Ltd., Beijing, China). For the specimen samples, the total DNAs were obtained from the Herbarium of Institute of Botany, the Chinese Academy of Sciences (PE), and the extraction method was according to Li, 2013. Then, 2 ×150 bp paired-end libraries were constructed and sequenced using an illumina NovaSeq 6000 platform (Illumina, San Diego, California, USA). The newly sequenced samples yielded around 6 Gbp of raw data. In order to assemble the draft genome of *Clematis*, we extracted total DNA from a *C. brevicaudata* DC. sample and constructed a library for sequencing, finally obtaining raw data of about 200 Gbp. All the genome skimming data were deposited in GenBank (**Supplementary Table S1**).

### Raw data processing

#### Plastid genome assembly

We used genome skimming data to assemble the complete plastid genome sequence using GetOrganelle v.1.7.5 (Jin et al., 2020). Detailed assembling process followed He et al. (2021). The assembled plastome sequences were annotated using Plann v.1.1.2 (Huang and Cronk, 2015) and manually adjusted by Geneious Prime v.2020 (Kearse et al., 2012).

#### Nuclear single-copy orthologous genes assembly using transcriptome data

Nuclear single-copy orthologs (SCOGs) were obtained from transcriptome data followed the pipeline of He et al. (2022). We used CD-HIT v.4.6.2 (Fu et al., 2012) to remove redundant sequences and TransDecoder v.5.0 (https://github.com/TransDecoder/TransDecoder/releases) to predict protein-coding regions. The assembly completeness of each sample was assessed using BUSCO v.5.2.2 (Simao et al., 2015). Subsequently, we constructed transcriptome homology scans using Proteinortho v.6.0.10 (Lechner et al., 2011) in the Diamond mode (Buchfink et al., 2015), and then searched the resulting clusters to identify gene families using a Python script “get_seq_from_proteinortho.py” (https://github.com/HeJian151004/get_seq_from_proteinortho). We then deleted all the organelle genome sequences from the SCOGs using the script “del_chloro_mito_from_fasta.py” (https://github.com/HeJian151004/del_chloro_mito_from_fasta), and used Treeshrink v.1.3.9 (Mai and Mirarab, 2018) to delete sequences that may be incorrectly clustered (showing unexpectedly long branches in the gene tree). Finally, we selected two SCOGs datasets with alignment length at least 1,000 bp (SCOG1000) and 3,000 bp (SCOG3000) for phylogenetic analysis.

#### Acquiring the nuclear SNPs data from genome skimming method

**Figure 1:**
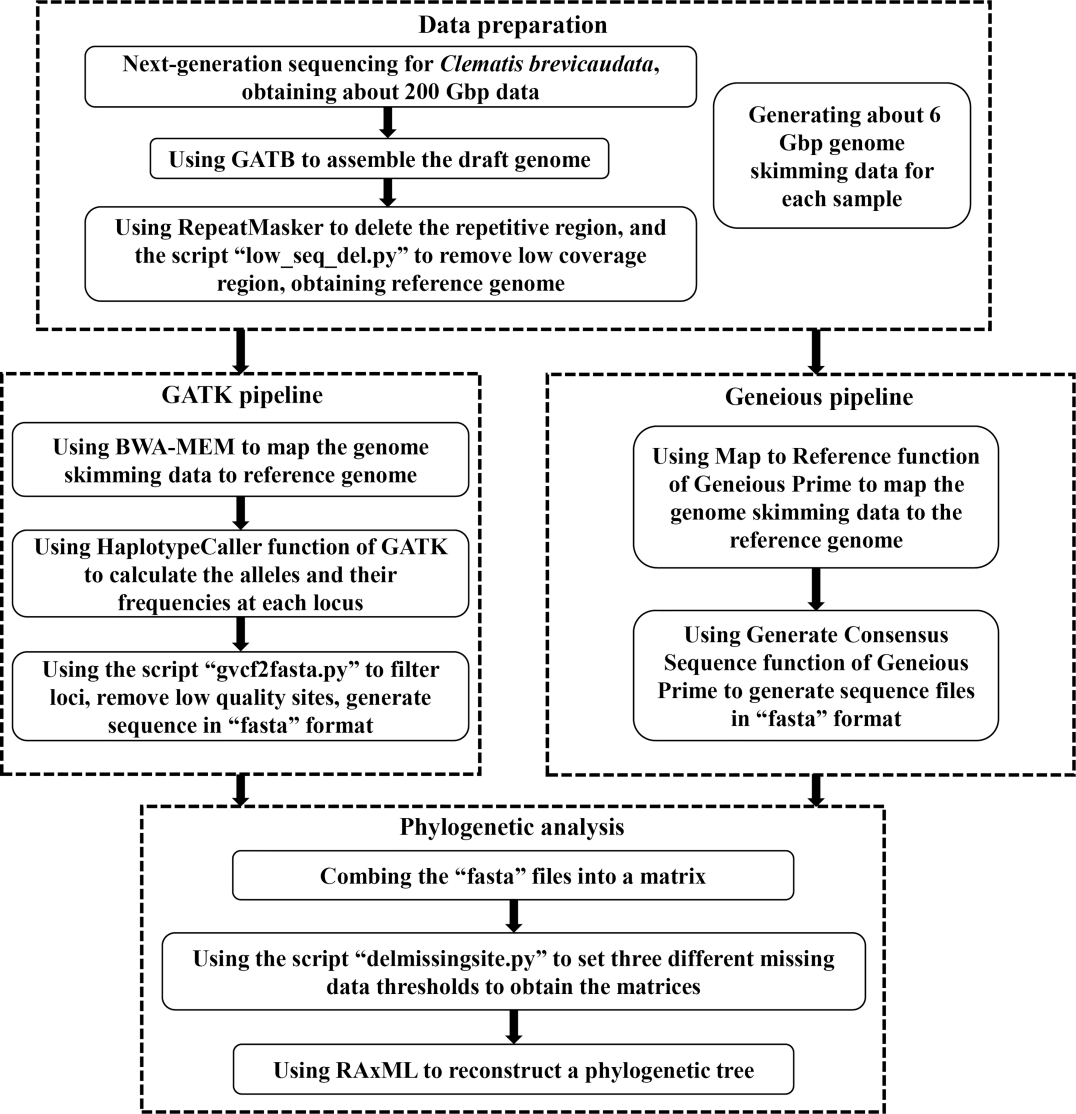
A flow chart of acquiring nuclear single nucleotide polymorphisms (SNPs) dataset from genome skimming data in this study.

For the genome skimming data, we further mined the nuclear SNPs data for phylogenetic inference. In brief, we assembled a draft genome as a reference, and then mapped the genome skimming data of other species to this reference genome to obtain the SNPs dataset. We used two methods to obtain the SNPs data, the GATK and the Geneious pipelines. Detailed process of both pipelines are as follows (also shown in **Figure 1**).

First, we used the GATB-Minia (https://github.com/GATB/gatb-minia-pipeline) to assemble the draft genome (Drezen et al., 2014). We obtained a draft genome of 7.81Gbp, which is too large to be applied for downstream analysis. Therefore, we used the RepeatMasker v.4.0.9 (Chen, 2004) to exclude the repetitive regions in the draft genome. We further deleted the low coverage regions by the following processes: the genome skimming data of five distantly related *Clematis* species [*C.leschenaultiana* DC., *C. repens* Finet et Gagnep., *C. songorica* Bunge, *C. tibetana* Kuntze, *C. viridis* (W. T. Wang and M. C. Chang) W. T. Wang] were mapped to the draft genome by Map to Reference function of Geneious Prime v.2020 (Kearse et al., 2012). Then we used a script “low_seq_del.py” (https://github.com/Jhe1004/low_seq_del) to remove the regions that none of the five samples were matched. After removing the duplicate and low coverage regions, we finally obtained a reference genome of 616 Mbp.

The GATK pipeline used BWA-MEM v.0.7.1 (Li, 2013) to map each genome skimming data back to the reference genome to generate “bam” format files. Then the HaplotypeCaller function of GATK v.4.2.5 (Mckenna et al., 2010) was applied to calculate the alleles and their frequencies at each locus. Then, GATK output the result as the “vcf” format file. Then, we used script “gvcf2fasta.py” (https://github.com/Jhe1004/gvcf2fasta) to convert “vcf” file to the “fasta” sequence. We filtered and deleted the site that met any of the following three criteria: (1) coverage less than 4, (2) site quality score less than 20, and (3) heterozygous.

The Geneious pipeline applied the Map to Reference function of Geneious Prime v.2020 (Kearse et al., 2012) to map the genome skimming data of each sample to the reference genome using Custom Sensitivity option with Allow Gaps off. Then we used the Generate Consensus Sequence function (using Trim to Reference Sequence option, and Most Common Bases for heterozygous sites) to generate sequence file of each sample, and finally saved these sequences as “fasta” files. All the alignments of this study, including the complete plastid genome sequences, SCOGs, and nuclear SNPs datasets, are deposited on Zenodo with the identifier https://doi.org/10.5281/zenodo.7215665.

### Phylogenetic analysis

Plastid genome structure and gene arrangement in *Clematis* species were checked according to the method of Liu et al. (2018), and then multiple sequence alignments were done using MAFFT v.7.471 (Katoh and Standley, 2013), after removing one inverted repeat (IR) region (He et al., 2019; He et al., 2021). We used both maximum likelihood (ML) and Bayesian inference (BI) methods for phylogenetic reconstruction. ML trees were generated by RAxML v.8.2.12 (Stamatakis, 2014) under the GTR+G model with bootstrap percentages computed after 100 replicates. BI analysis was performed using MrBayes v.3.2.3 (Ronquist et al., 2012) and the best substitution model (TVM+I+G) was tested by the AIC in jModelTest v.2.1.10 (Darriba et al., 2012). Markov chain Monte Carlo (MCMC) chains run 2,000,000 generations, sampling every 100 generations. The first 25% of the trees were discarded as burn-in, and the remaining trees were used to generate the consensus tree.

For the two SCOGs datasets, we applied both concatenation- and coalescent-based methods for phylogenetic reconstruction. For the concatenation method, genes of all the datasets were concatenated. Then, we used RAxML v.8.2.12 (Stamatakis, 2014) to reconstruct phylogeny with the GTR+G model and 100 replicates of bootstrap. For the coalescent-based method, single-gene trees were reconstructed by RAxML with the parameters as above. All gene trees were then inputted in ASTRAL v.4.4.4 (Zhang et al., 2018) for species tree inference.

The nuclear SNPs matrices obtained by both GATK and Geneious pipelines had a high proportion of missing data at many loci. Therefore, we set three missing data (percentage of gaps per alignment column, Duvall et al., 2020) thresholds for each pipeline and obtained six matrices: GATK-0.4MS, GATK-0.5MS, GATK-0.6MS (40%, 50%, and 60% missing data); Geneious-0MS, Geneious-0.05MS, Geneious-0.1MS (0, 5%, and 10% missing data). We used SNP-sites v.2.5.1 (Page et al., 2016) to remove the invariant sites. Then, all matrices were analyzed using ML method implemented in RAxML v.8.2.12 with “ASC_GTRGAMMA” model (Stamatakis, 2014) and 100 bootstrap replicates.

### Analysis of tree discordance

In this study, we explored the discordance among nuclear gene trees, between plastid and nuclear gene trees, and analyzed the possible biological causes. We tried to exclude factors such as sampling errors, stochastic errors, and paralogs (Zou and Ge, 2008), and tested the role of incomplete lineage sorting (ILS) and hybridization on the discordance of gene trees.

First, we examined the conflict among nuclear gene trees (the SCOG1000 dataset). In order to reduce the influence of stochastic error, gene trees with average support values more than 60 were chosen for analysis. We used Phyparts v.0.0.1 (Smith et al., 2015) to compare each nuclear gene tree with the species tree, calculated the proportion of gene trees concordant with the species tree at each node, and displayed them with pie charts. Meanwhile, to further visualize single-gene tree conflicts, we built cloud tree plots using the python package Toytree v.2.0.5 (Eaton, 2020).

The causes of nuclear gene tree conflicts were explored using a multiple species coalescent (MSC) model implemented in a simulation analysis to investigate whether ILS could be used to explain the conflict among nuclear gene trees (Yang et al., 2020; Morales-Briones et al., 2021). If the coalescent model fit the empirical gene trees well, the simulated gene trees would be consistent with the empirical gene trees, and ILS can explain the tree discordance. We used the function “sim.coaltree.sp” in the R package Phybase v.1.5 (Liu and Yu, 2010) to simulate 10,000 gene trees under the MSC model (the input coalescent species tree was constructed using the SCOG1000 dataset). Finally, we calculated the distances between each empirical gene tree and the species tree using DendroPy v.4.5.2 (Sukumaran and Holder, 2010), then showed the distance distribution between simulated gene trees and species tree using a histogram plot.

We also analyzed the causes of cyto-nuclear discordance and carried out a coalescent simulation study (Rose et al., 2021). We used the “sim.coaltree.sp” function in the R package Phybase v.1.5 (Liu and Yu, 2010) to simulate 10,000 gene trees, and then used PhyParts v.0.0.1 (Smith et al., 2015) to compare these simulated gene trees to the plastome phylogeny. If the discordant nodes are supported by a certain proportion of simulated gene trees, then it is probable that the conflict was caused by incomplete lineage sorting.

## Results

### Data of genome skimming, transcriptome, and draft genome

The genome skimming data size of each sample ranged from 5.07 Gbp (*C. songorica*) to 6.20 Gbp (*C. brevicaudata*), and the Q20 was 96.0%−98.9%, Q30 was 89.4%−96.9% (**Supplementary Table S1**). The data size of transcriptomes ranged from 5.41 Gbp (*C. viridis*) to 6.76 Gbp (*C. sibirica* Miller), and the Q20 was 97.3%−98.5%, Q30 was 93.0%−95.6%. The number of *de novo* transcripts varied from 53,429 (*C. tibetana*) to 147,758 (*C. macropetala* Ledeb.), and 37,188−114,171 transcripts were kept after removing redundancy. The N50 length of the transcripts ranged from 712 bp to 1,547 bp, and completeness of the assemblies comparing to BUSCO ranged from 54.2% (*C. reticulata* Walter) to 75.9% (*C. terniflora* DC.) (**Supplementary Table S2**). The size of *C. brevicaudata* genome draft was 7.81 Gbp with the contig N50 being 2,579 bp, and the number of contigs longer than 500 bp was 4,293,110 in total size of 6.19 Gbp.

### Plastid genome, nuclear SCOGs, nuclear SNPs data

We acquired a total of 32 *Clematis* plastome sequences ranging from 159,284 bp (*C. reticulata*) to 159,847 bp (*C. viridis*). The number and arrangement of the plastid genes of all the *Clematis* species are identical, all contained a pair of datIRs (31,023−31,082 bp.) separated by a large single copy region (79,074−79,693 bp) and a small single copy region (17,978−18,229 bp). All plastomes encoded a set of 112 genes, including 79 protein-coding genes, 29 transfer RNAs and four ribosomal RNAs (**Supplementary Table S3**). After removing IRa and poor alignment region, we finally obtained a matrix with aligned length of 128,149 bp for phylogenetic analysis.

For the transcriptome data, we obtained 9,900 SCOGs by homologous clusters after removing 106 organelle genes. We further discarded 3,782 genes, which were shorter than 1,000 bp, in subsequent analyses. Finally, the SCOG1000 dataset contained 6,118 genes (4,393 genes with average support value over 60), and SCOG3000 dataset contained 699 genes.

The data amount of nuclear SNPs matrix obtained by GATK and Geneious pipelines are different. The lengths of the matrices obtained by GATK pipeline are 21,767bp (GATK-0.4MS), 48,933 bp (GATK-0.5MS), and 100,223 bp (GATK-0.6MS), whereas those of the Geneious pipeline are 99,179 bp (Geneious-0MS), 375,536 bp (Geneious-0.05MS), and 2,066,289 bp (Geneious-0.1MS), respectively. The proportion of missing data in sect. *Naravelia* Prantl and sect. *Naraveliopsis* Hand.-Mazz. were significantly higher than those in other species. Because only 2.59 Gbp genome skimming data were available online for *C. fusca* Turcz. (**Supplementary Table S1**), high percentage of missing data was also present in its nuclear SNPs sequence.

### Phylogenetic analysis

#### Plastid phylogeny

**Figure 2:**
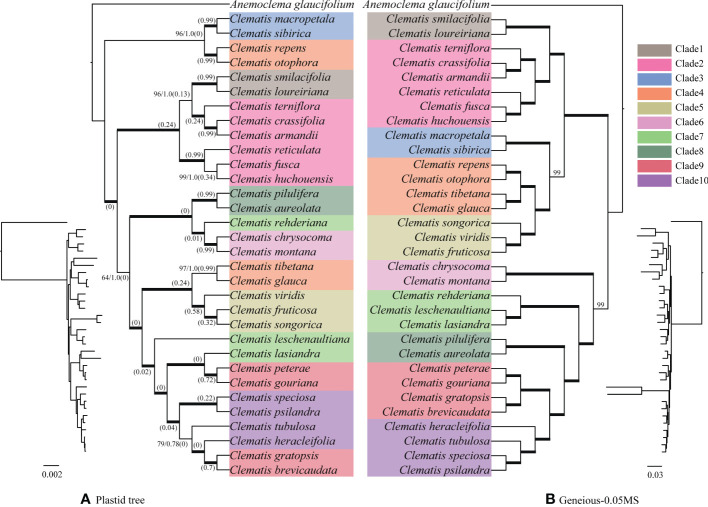
Bayesian phylogeny **(A)** of *Clematis* inferred from the plastid genome data and maximum likelihood phylogeny **(B)** inferred from nuclear SNPs of Geneious-0.05MS dataset. Cyto-nuclear conflicts are shown. In the plastid phylogeny **(A)**, bold branches show that the clades are 100% supported by both posteriori probability and ML bootstrap values. Otherwise, these two statistical values were marked on the branches. Numbers in brackets show the contribution of incomplete lineage sorting (ILS) to the conflicts between the simulated and plastid gene trees based on the multispecies coalescent model. Ten major clades were marked on the nuclear SNPs tree **(B)** with different colors. Species in plastid tree were marked with the same color with those in the nuclear SNPs tree.

For the plastid genome data, except a few clades with relatively weak support values, the majority of branches received full support (**Figure 2A**). Sect. *Naraveliopsis* Hand.-Mazz., sect. *Atragene* (L.) DC., sect. *Naravelia* (DC.) Prantl, sect. *Cheiropsis* DC., sect. *Meclatis* (Spach) Baillon, and sect. *Fruticella* Tamura (sensu Tamura, 1995) were shown to be monophyletic. Whereas, some sections, such as sect. *Campanella* Tamura, sect. *Clematis* (sensu Tamura, 1995), and sect. *Tubulosae* Decne. were not supported in the plastid phylogeny, and species of these sections were nested together.

#### Phylogeny of nuclear SCOGs

**Figure 3:**
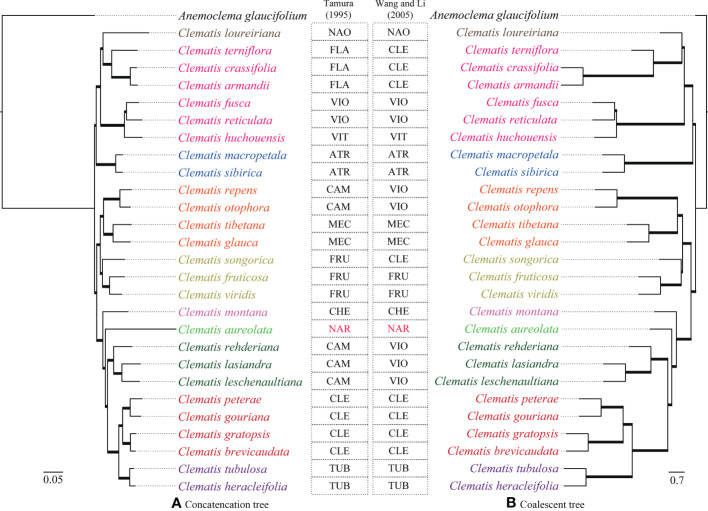
Phylogenetic trees inferred from SCOG1000 dataset by the concatenated (left) and the coalescence-based (right) methods. All clades of both trees are 100% supported and all the branches are in bold. Section abbreviations are: sect. *Atragene* (ATR), sect. *Naraveliopsis* (NAO), sect. *Clematis* (CLE), sect. *Flammula* (FLA), sect. *Viorna* (VIO), sect. *Viticella* (VIT), sect. *Campanella* (CAM), sect. *Meclatis* (MEC), sect. *Fruticella* (FRU), sect. *Cheiropsis* (CHE), sect. *Tubulosae* (TUB), *Naravelia* (NAR).

Two transcriptome-based datasets (SCOG1000 and SCOG3000) yielded highly congruent phylogenies in the coalescent-based and concatenated analyses (**Figure 3** and **Supplementary Figure S1**). All nodes in the SCOG1000 dataset using coalescent method obtained 100% support values. Whereas, in the SCOG3000 dataset, sect. *Fruticella* was not 100% supported, and the position of *C. songorica* was different in the coalescent and concatenated analyses (**Supplementary Figures S1**, **S2**). For the SCOG1000 dataset, except sect. *Campanella*, sect. *Viorna* (sensu Wang and Li, 2005) and sect. *Clematis* (sensu Wang and Li, 2005), which was shown to be polyphyletic, other sections were supported (**Figure 3**). Sect. *Clematis* (sensu Tamura, 1995) and sect. *Tubulosae* were both supported and tested to be sister groups

#### Nuclear SNPs phylogeny

The nuclear SNPs phylogenies based on the GATK pipeline were slightly different in basal branches which were insufficiently supported (**Supplementary Figures S3**). Among them, GATK-0.4MS dataset yielded a phylogeny which was more consistent with the trees inferred from the Geneious pipeline (**Supplementary Figure 4**). The resolved clades were also largely consistent with the SCOG1000 species tree (**Figure 3**). However, although sect. *Clematis* (sensu Tamura, 1995) and sect. *Tubulosae* showed close relationship in the three GATK datasets, the former section was shown to be paraphyletic to the latter (**Supplementary Figure S3**).

**Figure 4:**
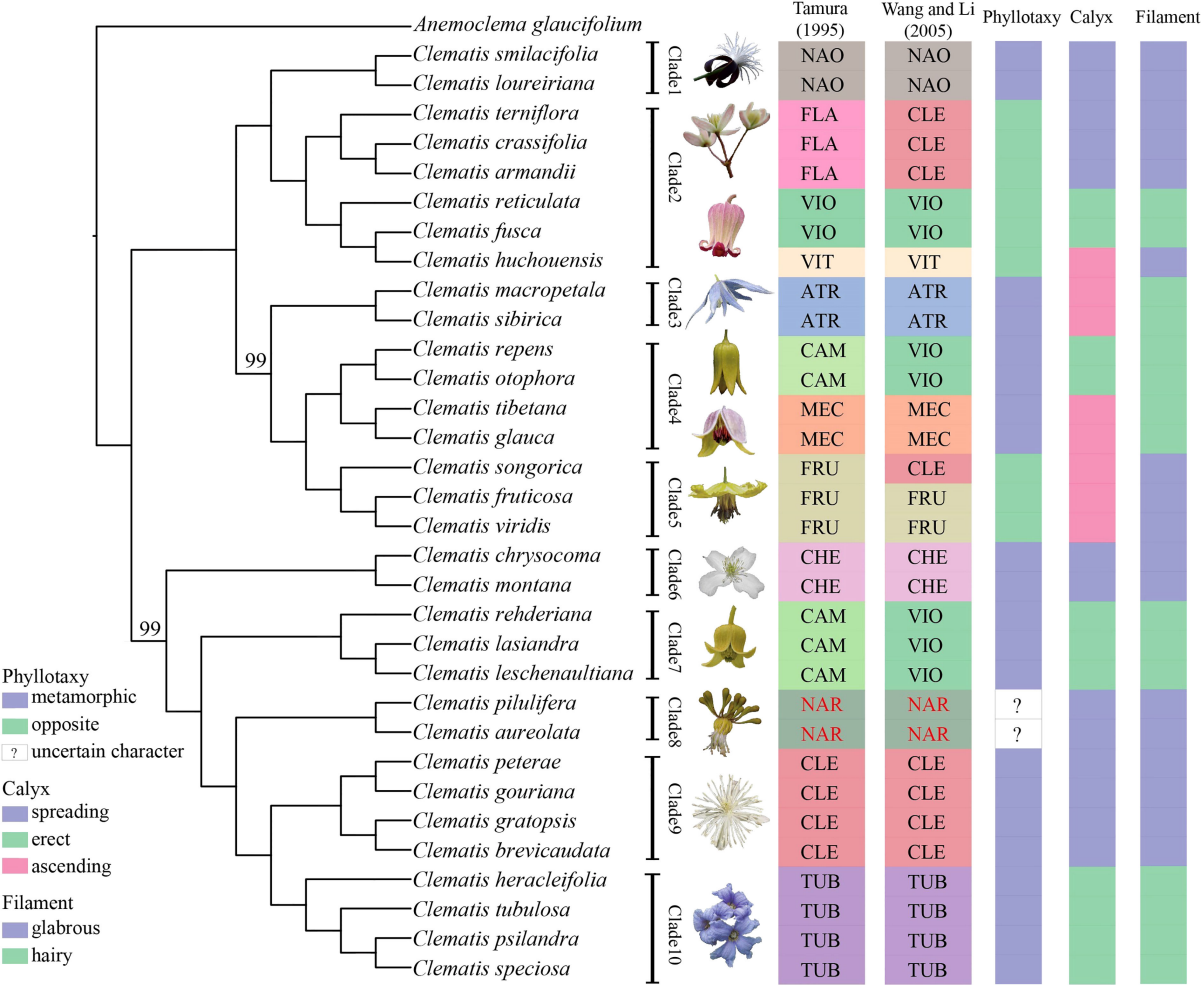
A maximum likelihood tree inferred form a nuclear SNPs dataset (Geneious-0.05MS). Two bootstrap values, which are less than 100, are marked above the branch, and all the other branches are fully supported. Section abbreviations follow **Figure 3**, and three important morphological characters are marked at right side of the tree.

The phylogenies inferred from the three datasets of Geneious pipeline were basically similar, but differed in support values (**Figure 4** and **Supplementary Figure S4**). The Geneious-0.05MS dataset produced the most robust phylogeny, which was almost the same with the SCOG1000 species tree. Their major difference was the position of sect. *Naraveliopsis* (**Figures 3**, **4**). Both SCOG1000 and the Geneious-0.05MS datasets had some well-supported incongruence with the plastid tree (**Figure 2**; **Supplementary Figure S2**). Because the nuclear SNPs dataset contains more samples than the SCOG1000 dataset, we discuss the phylogenetic relationships of *Clematis* mainly based on Geneious-0.05MS dataset (**Figure 4**). In this phylogenetic tree, ten major clades were resolved, and one section (sect. *Campanella*) in Tamura (1995) and two sections (sect. *Clematis* and sect. *Viorna*) in Wang and Li (2005) were shown to be polyphyletic. All the other sectional classification of the two systems were supported.

### Gene conflict analyses

**Figure 5:**
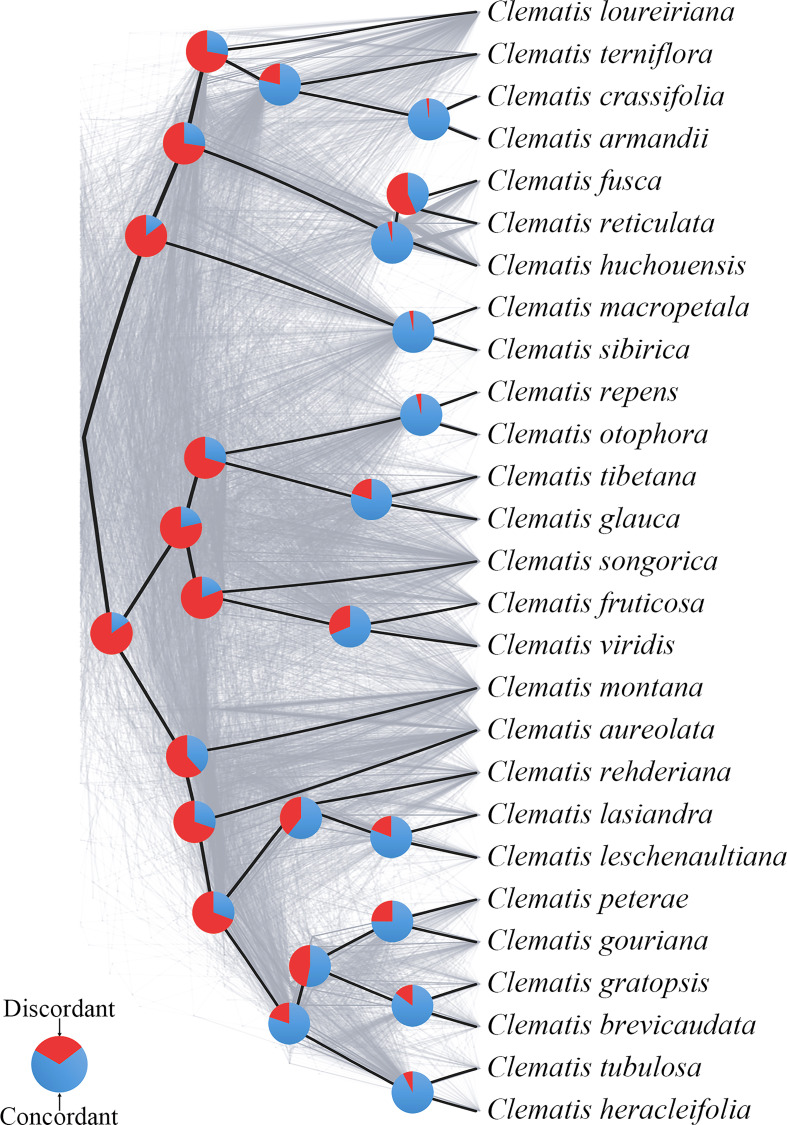
A cloud tree showing discordance among nuclear genes. The ASTRAL species tree (based on trees from SCOG1000 dataset with average bootstrap value more than 60) is in heavy black lines. All the branches are fully supported. The gray-colored trees (cloud tree) were sampled from 695 SCOGs (without missing taxa). Pie charts show the proportions of concordant and discordant topologies of gene trees comparing to the species tree.

**Figure 6:**
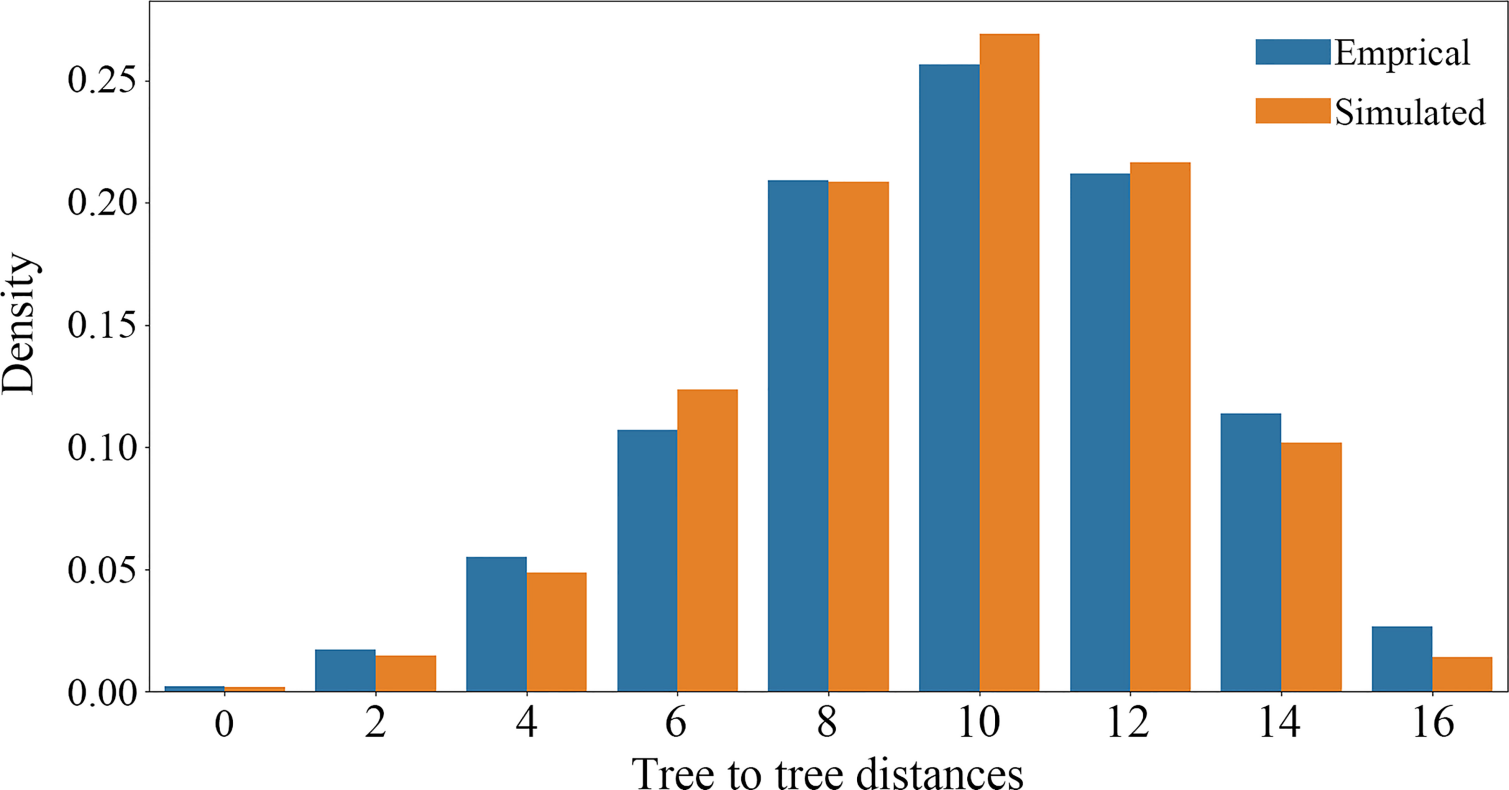
Coalescent simulations of tree-to-tree distance distributions between the ASTRAL species tree and the 4393 empirical (orange boxes) gene trees (based on trees from SCOG1000 dataset with average bootstrap value more than 60) and those from the 10,000 simulation trees (blue boxes).

Using SCOG1000 (and average bootstrap value more than 60) dataset, high levels of gene tree discordances were detected mainly at deep nodes (**Figure 5**). Coalescent simulation analysis (**Figure 6**) showed similar pattern between empirical and simulated distance distributions, indicating that ILS alone can explain most of the gene tree conflicts. However, the contradiction between some nodes of the plastid and the nuclear species trees cannot be explained by ILS (**Figure 2A**). For example, species of Clade 9 (sect. *Clematis* sensu Tamura, 1995) and Clade 10 (sect. *Tubulosae*) (**Figure 2B**) were clustered together in the plastome phylogeny (**Figure 2A**), and Phyparts result showed no simulated gene trees were concordant to the empirical plastome tree. Moreover, some species of sect. *Campanella* (such as *C. rehderiana*) also showed different positions in the simulated gene trees and the plastome tree, suggesting that ILS can be excluded for explaining its cyto-nuclear discordance, and hybridization and introgression might be the main cause.

## Discussion

### Phylogenomic data for *Clematis*

Seed plants encompass a high level of diversity of genome size varying by more than 2,000-fold (Novák et al., 2020). Larger genomes generally contain more proportion of repeat sequences, transposable elements, and other non-transcribed low-copy sequences, while the amount of expressed genes are rather stable with about 0.03 Gbp (Kersey, 2019). For this reason, we do not need to consider the plant genome size when choosing transcriptome method for phylogenetic studies. However, when choosing genome skimming data, the size of plant genome becomes a vital issue that should be considered.

*Clematis* species have large genome size, which makes high-depth sequencing (10 × or more) unaffordable, and low-depth genome skimming data (less than 1 ×) of *Clematis* have been only used for assembling the plastome sequences or tandemly repeat nrDNA regions which have high copy numbers in the genome (He et al., 2021). The plastome phylogeny of *Clematis* (He et al., 2021) have better resolved the relationships within the genus than those of the Sanger sequencing data (e.g., Miikeda et al., 2006; Xie et al., 2011; Lehtonen et al., 2016). However, there were still some major clades with weak support and some clades are unexplainable taxonomically. The nrDNA sequences also failed to generate a robust tree due to insufficient phylogenetic information (He et al., 2021).

Although plastome data have been successfully used for phylogenetic reconstruction of plant taxa at almost all taxonomic levels, studies have shown that the plastome data alone may sometimes not sufficiently resolve the phylogeny of closely related species due to frequent hybridization and introgression in plants (Liu et al., 2022). Therefore, care should be taken when using plastome data alone to resolve species relationships of plant taxa. This is also the case with *Clematis*. Evidences from horticulture (Yuan et al., 2010), molecular phylogenetic studies (Lyu et al., 2021), and the present study showed that there is widespread hybridization among *Clematis* species or even between sections. In this study, transcriptome data were successfully assembled with thousands of SCOGs which robustly resolved the phylogenetic framework of *Clematis*. The SCOG1000 dataset not only fully resolved *Clematis* phylogeny but also provided a tree that corresponded well to morphological groups. The RNA-seq method is easy, fast, efficient for acquiring highly reusable nuclear genome data, independent of plant genome size (Cheon et al., 2020), and maybe the best choice for phylogenetic study of *Clematis* so far. The major problem with transcriptome method is that it cannot be successfully applied for herbarium materials. If we want to include more herbarium samples, data partitioning method should be reconsidered.

Previous studies have used genome skimming data to obtain nuclear SNPs by mapping reads to the reference genome (Olofsson et al., 2019; Zhang et al., 2019). This study presented a further exploration of this method in *Clematis*. The phylogenies from the nuclear SNPs data by the two pipelines in this study were better resolved than the previous published nrDNA tree (He et al., 2021). Two different pipelines generated different amounts of data, and Geneious pipeline produced larger datasets than GATK pipeline. In the same way, Geneious pipeline generated more robust phylogeny which was almost the same with that reconstructed by SCOGs. Meanwhile, two herbarium samples (*C. psilandra* and *C. speciosa*) and four genome skimming data (*C. fusca*, *C. macropetala*, *C. pilulifera*, and *C. reticulata*) from other studies with lower sequencing depth clustered in the correct positions on the nuclear SNPs tree (**Figure 4**). Therefore, this method (especially the Geneious pipeline) is reliable and may play an important role in future phylogenetic study of *Clematis* with comprehensive sampling.

The problems of this method, however, also need to be mentioned. Because the sequencing depth is low, SNP genotyping and allele frequency estimation may be biased by those genome skimming data. So, the SNPs datasets may not be applied for population genetic analysis, such as STRUCTURE (Pritchard et al., 2000). Furthermore, this data may also not work well for analysis of reticulate evolution (such as HyDe, Blischak et al., 2018) and whole genome duplication detection (WGD, Yang et al., 2019).

### Phylogenetic inferences of *Clematis*

Although previous phylogenetic studies used more samples (Xie et al., 2011; Lehtonen et al., 2016), insufficient resolution by small number of DNA regions has hindered our understanding of the evolution of *Clematis*. The plastome data took us a step forward in resolving the phylogeny of the genus (He et al., 2021). Plastome phylogeny, inferred by He et al. (2021), resolved six major clades in *Clematis*. Except a clade comprising only species of sect. *Naravelia*, all the other five clades contained three or more sections. Despite the smaller sample size of this study, all the six corresponding clades were also resolved by our plastome phylogenetic analysis. These clades (except sect. *Naravelia* clade) were difficult to be defined by morphology.

In this study, using nuclear SNPs and SCOG data, we reconstructed the first well resolved phylogenetic backbone of *Clemati*s. Most of the morphologically defined sections were supported. Trees inferred from the nuclear genome data (**Figures 2**–**4**) were better corresponding to morphological characters than plastome phylogeny. The Geneious-0.05MS dataset resolved ten major clades in *Clematis* (**Figures 2**, **4**). Clade 1 represents subtropical sect. *Naraveliopsis* which has conspicuous connective projections on the anthers. Clade 2 comprises species of sect. *Flammula* DC., sect. *Viticella* DC. and sect. *Viorna* (sensu Tamura, 1995). The synapomorphy may be their type II seedlings (or opposite seedling leaves, Essig, 1991). Clade 3 represents sect. *Atragene*, which has petal-like staminodes in the flowers. Clade 4, including sect. *Meclatis* and species of sect. *Campanella* with yellow flowers and hairy filaments and anthers, is characterized by its yellow and thick sepals. Clade 5 represents sect. *Fruticella* with erect shrubby stem. Clade 6 represents sect. *Cheiropsis*, which is characterized by its flowers arising from old or hornotinous branches. Clade 7 contains some species of sect. *Campanella*. Their shared characteristics are the type I seedling (or alternate seedling leaves, Essig, 1991), erect sepals, hairy stamen filaments and glabrous anthers. Clade 8 is sect. *Naravelia* which was recognized as a distinct genus by Tamura (1995) and Wang and Li (2005). Plants of this section possess leaf tendrils and spoon-shaped petals. Clade 9 represents the narrowly defined sect. *Clematis* (sensu Tamura, 1995), which is characterized by the type I seedling, small white flowers, spreading sepals, and glabrous stamens. Clade 10 represents sect. *Tubulosae*, which is characterized by the type I seedling, ternate leaves, erect herbaceous stem, erect sepals and hairy stamens. It should be pointed out that the range of our sample was relatively narrow, and future studies with more comprehensive sampling are needed to further elucidate the phylogenetic and taxonomic problems in *Clematis*.

Using nuclear genome data, we also gained new knowledge and insights into some taxonomic issues for *Clematis* in this study. Previous studies have suggested that sect. *Campanella* may be a polyphyletic group (He et al., 2021). Our nuclear genome phylogeny confirmed that *C. repens* and *C. otophora* (in sect. *Campanella*) are more closely related to sect. *Meclatis* (clade 4, **Figure 4**) rather than to other sect. *Campanella* species. Both *C. repens* and *C. otophora* have yellow flowers with thick sepals which are more similar to those of the sect. *Meclatis*. Two morphologically well diverged sections, sect. *Clematis* (sensu Tamura, 1995) and sect. *Tubulosae*, have shown to be very closely related or even cannot be clearly separated by Sanger sequencing data (Xie et al., 2011; Lehtonen et al., 2016; Yan et al., 2016). They were also nested together in our plastome tree (**Figure 2**), but were clearly separated by our SCOG1000 data (**Figure 3**) and Geneious-0.05MS data (clade 9 and clade 10, **Figures 2**–**4**). The simulation results showed that this cyto-nuclear discordance may be caused by hybridization events between the two sections (**Figure 2A**). Hybridization events between these two morphologically diverged sections have been also confirmed by other reports, horticultural evidence, and phylogenomic analysis (Makino, 1907; Yuan et al., 2010; Lyu et al., 2021).

Similar to other studies, our results showed that all the important morphological characters emphasized by taxonomists, such as phyllotaxy, calyx, and filament hairs (Tamura, 1995), may have evolved multiple times, and it is difficult to make subgeneric classification by using a few key characters. Specifically, we emphasis that seedling morphology (phyllotaxy as in this study), highlighted by Tamura (1995), should be based on observations but not speculation. Majority number of *Clematis* species have no real observation data of seedling morphology. Seedling status of many sections (such as sect. *Naraveliopsis* and sect. *Fruticella*) proposed by Tamura (1995) are likely to be wrong (Cheng et al., 2016). Based on our observation, seedling morphology of sect. *Fruticella* (not published) should be type I (metamorphic, Essig, 1991) and similar to that of sect. *Meclatis* (rather than type II proposed by Tamura, 1995). So, before using seedling morphology for taxonomic treatment, this character needs to be studied through comprehensive observation.

Our findings also shed light on the evolutionary history of *Clematis*. Studies have shown that *Clematis* may have experienced recent species radiation during the late Neogene and the Quaternary (Xie et al., 2011; He et al., 2021). Recent species radiation may lead to severe lineage sorting when the ancestral population was large (Pamilo and Nei, 1988), and this fits well with our simulation results (**Figures 5**, **6**). Our results demonstrated that there are extensive gene tree conflicts at early diverged nodes, which can be explained by ILS. Meanwhile, our analysis of cyto-nuclear discordance (**Figure 2**) suggested that there may also have been widespread interspecific hybridization events in *Clematis*, which contributed to high level of incongruence between plastid and nuclear phylogenies. From our analysis, both ILS and interspecific hybridization in *Clematis* made its classification and phylogenetic analysis very difficult, especially using small number of DNA regions or plastome data alone.

### Consideration of genome partitioning selection for other plant taxa

There are several other genera in Ranunculaceae that are similar to *Clematis*, such as *Anemone* L., *Aconitum* L., and *Delphinium* L. These genera have not only large genome size (https://cvalues.science.kew.org/search) but also have hundreds of wild species (Tamura, 1995). In addition, they all have no high-quality whole genome reference available and few phylogenomic studies with comprehensive sampling. Resolving the phylogenetic framework of those taxa is highly possible to encounter the same conditions with *Clematis*: ineffectiveness of Sanger sequencing data and difficulty in genome partitioning selection. Furthermore, studies have shown that the plastid genome (or regions) data alone did not work well for the phylogenetic reconstruction of those taxa (Hoot et al., 2012; Jiang et al., 2017; Hong et al., 2017; Xiang et al., 2017). Our results suggested that transcriptome method may be the first choice for solving the problem, and if the samples are not suitable for RNA extraction, Geneious pipeline presented in this study (using low-depth genome skimming data) can be tried. Although this study did not test target enrichment data, this method is also recommended if the complicated experimental procedures are acceptable to the researchers.

Genome size may be an important factor in genome partitioning selection. If the genome size of concerning taxon is small (less than 1 Gbp), genome skimming method can easily obtain high sequencing depth at an acceptable cost, and is a good choice to solve phylogenetic problems. We have tried to obtain and successfully assembled SCOGs (not published) from 6 Gbp of genome skimming data from *Epilobium* L. (Onagraceae) samples using the method of Liu et al. (2021). The genome size of *Epilobium* species is about 0.2 Gbp, and our data was up to 30 × in sequencing depth. In this case, genome skimming method is better than transcriptome and target enrichment method. Using this data, we can acquire the plastome and nuclear SCOGs data from both transcribed region and non-transcribed (intron, spacer, repetitive regions, and so on) regions, and conduct a variety of downstream analysis, such as phylogenetic reconstruction, molecular dating, hybridization analysis, and WGD detection.

## Data availability statement

The data presented in the study are deposited in the NCBI BioProject database (https://www.ncbi.nlm.nih.gov/bioproject/), accession number PRJNA838588 and PRJNA776151.

## Author contributions

JX, RL, and JH, analyzed the data and prepared the draft. ML, JH, JJ, and LX conducted the sample gathering. JH, JC, and LX designed the study. JX, RL, and LX wrote and revised the manuscript. All the authors contributed to the article and approved the submitted version.

## Funding

This study was supported by the National Natural Science Foundation of China (grant numbers 32270223, 31670207).

## Acknowledgments

We thank Ma Xin-Tang and Ban Qin, working in the Herbarium of Institute of Botany, the Chinese Academy of Sciences (PE), for kindly providing *Clematis* specimen samples. We are grateful to Dr. Xu Chao from Institute of Botany, the Chinese Academy of Sciences for extracting high quality DNAs from specimen samples for this study.

## Conflict of interest

The authors declare that the research was conducted in the absence of any commercial or financial relationships that could be construed as a potential conflict of interest.

## Publisher’s note

All claims expressed in this article are solely those of the authors and do not necessarily represent those of their affiliated organizations, or those of the publisher, the editors and the reviewers. Any product that may be evaluated in this article, or claim that may be made by its manufacturer, is not guaranteed or endorsed by the publisher.

## Supplementary material

Supplementary Table S1 Sample information of genome skimming data in this study.

Supplementary Table S2 Sample information of transcriptome data in this study.

Supplementary Table S3 Plastid genome features of the Clematis species in this study.

Supplementary Figure 1 Phylogenetic trees constructed by concatenated and coalescence-based methods based on SCOG3000 dataset.

Supplementary Figure 2 Bayesian phylogeny **(A)** of *Clematis* inferred from the plastid genome data and maximum likelihood phylogeny **(B)** inferred from of SCOG1000 data. Cyto-nuclear discordance is shown.

Supplementary Figure 3 Maximum likelihood phylogenetic trees constructed from three nuclear SNPs data matrices obtained by GATK pipeline.

Supplementary Figure 4 Maximum likelihood phylogenetic trees constructed from two nuclear SNPs data matrices obtained by Geneious pipeline.

## References

[B1] Blischak P. D. Chifman J. Wolfe A. D. Kubatko L. S. (2018). HyDe: A Python package for genome-scale hybridization detection. Syst. Biol. 67, 821–829. doi: 10.1093/sysbio/syy023 29562307 PMC6454532

[B2] Buchfink B. Xie C. Huson D. (2015). Fast and sensitive protein alignment using DIAMOND. Nat. Methods 12, 59–60. doi: 10.1038/nmeth.3176 25402007

[B3] Chen N. S. (2004). Using repeat masker to identify repetitive elements in genomic sequences. Curr. Protoc. Bioinf. 5, 4–10. doi: 10.1002/0471250953.bi0410s05 18428725

[B4] Cheng J. Yan S. X. Liu H. J. Lin L. L. Li J. Y. Liao S.. (2016). Reconsidering the phyllotaxy significance of seedlings in *Clematis* . Phytotaxa 265, 131–138. doi: 10.11646/phytotaxa.265.2.4

[B5] Chen S. F. Zhou Y. Q. Chen Y. R. Gu J. (2018). Fastp: An ultra-fast all-in-one FASTQ preprocessor. Bioinformatics 34, i884–i890. doi: 10.1093/bioinformatics/bty560 30423086 PMC6129281

[B6] Cheon S. Zhang J. Park C. Teeling E. (2020). Is phylotranscriptomics as reliable as phylogenomics? Mol. Biol. Evol. 37, 3672–3683. doi: 10.1093/molbev/msaa181 32658973 PMC7743905

[B7] Darriba D. Taboada G. Doallo R. Posada D. (2012). jModelTest 2: more models, new heuristics and parallel computing. Nat. Methods 9, 772. doi: 10.1038/nmeth.2109 PMC4594756 22847109

[B8] Dodsworth S. (2015). Genome skimming for next-generation biodiversity analysis. Trends Plant Sci. 20, 525–527. doi: 10.1016/j.tplants.2015.06.012 26205170

[B9] Drezen E. Rizk G. Chikhi R. Deltel C. Lemaitre C. Peterlongo P.. (2014). GATB: genome assembly & analysis tool box. Bioinformatics 30, 2959–2961. doi: 10.1093/bioinformatics/btu406 24990603 PMC4184257

[B10] Duvall M. R. Burke S. V. Clark D. C. (2020). Plastome phylogenomics of poaceae: Alternate topologies depend on alignment gaps. Bot. J. Linn. Soc 192, 9–20. doi: 10.1093/botlinnean/boz060

[B11] Eaton D. A. R. (2020). Toytree: A minimalist tree visualization and manipulation library for Python. Methods Ecol. Evol. 11, 187–191. doi: 10.1111/2041-210X.13313

[B12] Essig F. B. (1991). Seedling morphology in *Clematis* (Ranunculaceae) and its taxonomic implications. SIDA 1991, 377–390.

[B13] Fonseca L. H. M. Lohmann L. G. (2020). Exploring the potential of nuclear and mitochondrial sequencing data generated through genome-skimming for plant phylogenetics: A case study from a clade of neotropical lianas. J. Syst. Evol. 58, 18–32. doi: 10.1111/jse.12533

[B14] Fu L. M. Niu B. F. Zhu Z. W. Wu S. T. Li W. Z. (2012). CD-HIT: Accelerated for clustering the next-generation sequencing data. Bioinformatics 28, 3150–3152. doi: 10.1093/bioinformatics/bts565 23060610 PMC3516142

[B15] Grabherr M. G. Haas B. J. Yassour M. Levin J. Z. Thompson D. A. Amit I.. (2011). Full-length transcriptome assembly from RNA-seq data without a reference genome. Nat. Biotechnol. 29, 644–652. doi: 10.1038/nbt.1883 21572440 PMC3571712

[B16] Grey-Wilson C. (2000). Clematis, the genus (Portland, OR: Timber Press).

[B17] He J. Lyu R. D. Luo Y. K. Lin L. L. Yao M. Xiao J. M.. (2021). An updated phylogenetic and biogeographic analysis based on genome skimming data reveals convergent evolution of shrubby habit in *Clematis* in the pliocene and pleistocene. Mol. Phylogenet. Evol. 164, 107259. doi: 10.1016/j.ympev.2021.107259 34303792

[B18] He J. Lyu R. D. Luo Y. K. Xiao J. M. Xie L. Wen J.. (2022). A phylotranscriptome study using silica gel-dried leaf tissues produces an undated robust phylogeny of ranunculaceae. Mol. Phylogenet. Evol. 174, 107545. doi: 10.1016/j.ympev.2022.107545 35690374

[B19] He J. Yao M. Lyu R. D. Lin L. L. Liu H. J. Pei L. Y.. (2019). Structural variation of the complete chloroplast genome and plastid phylogenomics of the genus *Asteropyrum* (Ranunculaceae). Sci. Rep. 9, 1–13. doi: 10.1038/s41598-019-51601-2 31653891 PMC6814708

[B20] Hong Y. Luo Y. Gao Q. Ren C. Yuan Q. Yang Q. E. (2017). Phylogeny and reclassification of *Aconitum* subgenus *Lycoctonum* (Ranunculaceae). PLoS One 12, e0171038. doi: 10.1371/journal.pone.0171038 28141851 PMC5334035

[B21] Hoot S. B. Meyer K. M. Manning J. C. (2012). Phylogeny and reclassification of *Anemone* (Ranunculaceae), with an emphasis on austral species. Syst. Bot. 37, 139–152. doi: 10.1600/036364412X616729

[B22] Huang D. I. Cronk Q. C. B. (2015). Plann: A command–line application for annotating plastome sequences. Appl. Plant Sci. 3, 1500026. doi: 10.3732/apps.1500026 PMC4542940 26312193

[B23] Jiang N. Zhou Z. Yang J. B. Zhang S. D. Guan K. Y. Tan Y. H.. (2017). Phylogenetic reassessment of tribe anemoneae (Ranunculaceae): Non-monophyly of *Anemone s. l.* revealed by plastid datasets. PLoS One 12, e0174792. doi: 10.1371/journal.pone.0174792 28362811 PMC5376084

[B24] Jin J. J. Yu W. B. Yang J. B. Song Y. dePamphilis C. W. Yi T. S.. (2020). GetOrganelle: A fast and versatile toolkit for accurate *de novo* assembly of organelle genomes. Genome Biol. 21, 241. doi: 10.1186/s13059-020-02154-5 32912315 PMC7488116

[B25] Johnson M. (1997). Släktet klematis (Södertälje: Magnus Johnson Plantskola).

[B26] Katoh K. Standley D. M. (2013). MAFFT multiple sequence alignment software version 7: improvements in performance and usability. Mol. Biol. Evol. 30, 772–780. doi: 10.1093/molbev/mst010 23329690 PMC3603318

[B27] Kearse M. Moir R. Wilson A. Stones-Havas S. Cheung M. Sturrock S.. (2012). Geneious basic: An integrated and extendable desktop software platform for the organization and analysis of sequence data. Bioinformatics 28, 1647–1649. doi: 10.1093/bioinformatics/bts199 22543367 PMC3371832

[B28] Kersey P. J. (2019). Plant genome sequences: Past, present, future. Curr. Opin. Plant Biol. 48, 1–8. doi: 10.1016/j.pbi.2018.11.001 30579050

[B29] Khan G. Nolzen J. Schepker H. Albach D. C. (2021). Incongruent phylogenies and their implications for the study of diversification, taxonomy, and genome size evolution of *Rhododendron* . Am. J. Bot. 108, 1957–1981. doi: 10.1002/ajb2.1747 34668570

[B30] Kress W. J. Soltis D. E. Kersey P. J. Soltis P. S. (2022). Green plant genomes: What we know in an era of rapidly expanding opportunities. Proc. Natl. Acad. Sci. U.S.A. 119, e2115640118. doi: 10.1073/pnas.2115640118 35042803 PMC8795535

[B31] Lechner M. Findeiß S. Steiner L. Marz M. Stadler P. F. Prohaska S. J. (2011). Proteinortho: detection of (co-) orthologs in large-scale analysis. BMC Bioinf. 12, 1–9. doi: 10.1186/1471-2105-12-124 PMC3114741 21526987

[B32] Lee-Yaw J. A. Grassa C. J. Joly S. Andrew R. L. Rieseberg L. H. (2019). An evaluation of alternative explanations for widespread cytonuclear discordance in annual sunflowers (*Helianthus*). New Phytol. 221, 515–526. doi: 10.1111/nph.15386 30136727

[B33] Lehtonen S. Christenhusz M. J. M. Falck D. (2016). Sensitive phylogenetics of *Clematis* and its position in ranunculaceae. Bot. J. Linn. Soc 182, 825–867. doi: 10.1111/boj.12477

[B34] Li H. (2013). Aligning sequence reads, clone sequences and assembly contigs with BWA-MEM. arXiv 1303, 3997. doi: 10.48550/arXiv.1303.3997

[B35] Li H. T. Luo Y. Gan L. Ma P. F. Gao L. M. Yang J. B.. (2021). Plastid phylogenomic insights into relationships of all flowering plant families. BMC Biol. 19, 232. doi: 10.1186/s12915-021-01166-2 34711223 PMC8555322

[B36] Liu H. J. He J. Ding C. H. Lyu R. D. Pei L. Y. Cheng J.. (2018). Comparative analysis of complete chloroplast genomes of *Anemoclema*, *Anemone*, *Pulsatilla*, and *Hepatica* revealing structural variations among genera in tribe anemoneae (Ranunculaceae). Front. Plant Sci. 9. doi: 10.3389/fpls.2018.01097 PMC6073577 30100915

[B37] Liu B. B. Hong D. Y. Zhou S. L. Xu C. Dong W. P. Johnson G.. (2019). Phylogenomic analyses of the *Photinia* complex support the recognition of a new genus *Phippsiomeles* and the resurrection of a redefined *Stranvaesia* in maleae (Rosaceae). J. Syst. Evol. 57, 678–694. doi: 10.1111/jse.12542

[B38] Liu B. B. Ma Z. Y. Ren C. Hodel R. G. J. Sun M. Liu X. Q.. (2021). Capturing single-copy nuclear genes, organellar genomes, and nuclear ribosomal DNA from deep genome skimming data for plant phylogenetics: A case study in vitaceae. J. Syst. Evol. 59, 1124–1138. doi: 10.1111/jse.12806

[B39] Liu B. B. Ren C. Kwak M. Hodel R. G. J. Xu C. He J.. (2022). Phylogenomic conflict analyses in the apple genus *Malus s. l.* reveal widespread hybridization and allopolyploidy driving diversification, with insights into the complex biogeographic history in the northern hemisphere. J. Integr. Plant Biol. 64, 1020–1043. doi: 10.1111/jipb.13246 35274452

[B40] Liu L. Yu L. (2010). Phybase: An r package for species tree analysis. Bioinformatics 26, 962–963. doi: 10.1093/bioinformatics/btq062 20156990

[B41] Li J. L. Wang S. Yu J. Wang L. Zhou S. L. (2013). A modified CTAB protocol for plant DNA extraction. Chin. Bull. Bot. 48, 72–78. doi: 10.3724/SP.J.1259.2013.00072

[B42] Li H. T. Yi T. S. Gao L. M. Ma P. F. Zhang T. Yang J. B.. (2019). Origin of angiosperms and the puzzle of the Jurassic gap. Nat. Plants 5, 461–470. doi: 10.1038/s41477-019-0421-0 31061536

[B43] Lyu R. D. He J. Luo Y. K. Lin L. L. Yao M. Cheng J.. (2021). Natural hybrid origin of the controversial “species” *Clematis × pinnata* (Ranunculaceae) based on multidisciplinary evidence. Front. Plant Sci. 12. doi: 10.3389/fpls.2021.745988 PMC8545901 34712260

[B44] Mai U. Mirarab S. (2018). TreeShrink: fast and accurate detection of outlier long branches in collections of phylogenetic trees. BMC Genomics 19, 272. doi: 10.1186/s12864-018-4620-2 29745847 PMC5998883

[B45] Makino T. (1907). Observations on the flora of Japan. Bot. Magaz. (Tokyo) 21, 86–88.

[B46] Marks R. A. Hotaling S. Frandsen P. B. VanBuren R. (2021). Representation and participation across 20 years of plant genome sequencing. Nat. Plants 7, 1571–1578. doi: 10.1038/s41477-021-01031-8 34845350 PMC8677620

[B47] McKain M. R. Johnson M. G. Uribe-Convers S. Eaton D. Yang Y. (2018). Practical considerations for plant phylogenomics. Appl. Plant Sci. 6, e1038. doi: 10.1002/aps3.1038 29732268 PMC5895195

[B48] Mckenna A. Hanna M. Banks E. Sivachenko A. Cibulskis K. Kernytsky A.. (2010). The genome analysis toolkit: A map reduce framework for analyzing next-generation DNA sequencing data. Genome Res. 20, 1297–1303. doi: 10.1101/gr.107524.110 20644199 PMC2928508

[B49] Miikeda O. Kita K. Handa T. Yukawa T. (2006). Phylogenetic relationships of *Clematis* (Ranunculaceae) based on chloroplast and nuclear DNA sequences. Bot. J. Linn. Soc 152, 153–168. doi: 10.1111/j.1095-8339.2006.00551.x

[B50] Morales-Briones D. F. Kadereit G. Tefarikis D. T. Moore M. J. Smith S. A. Brockington S. F.. (2021). Disentangling sources of gene tree discordance in phylogenomic data sets: Testing ancient hybridizations in amaranthaceae s. l. Syst. Biol. 70, 219–235. doi: 10.1093/sysbio/syaa066 32785686 PMC7875436

[B51] Novák P. Guignard M. S. Neumann P. Kelly L. J. Mlinarec J. Koblížková A.. (2020). Repeat-sequence turnover shifts fundamentally in species with large genomes. Nat. Plants 6, 1325–1329. doi: 10.1038/s41477-020-00785-x 33077876

[B52] Olofsson J. K. Cantera I. Van de Paer C. Hong-Wa C. Zedane L. Dunning L. T.. (2019). Phylogenomics using low-depth whole genome sequencing: A case study with the olive tribe. Mol. Ecol. Resour. 19, 877–892. doi: 10.1111/1755-0998.13016 30934146

[B53] One Thousand Plant Transcriptomes Initiative (2019). One thousand plant transcriptomes and the phylogenomics of green plants. Nature 574, 679–685. doi: 10.1038/s41586-019-1693-2 31645766 PMC6872490

[B54] Page A. J. Taylor B. Delaney A. J. Soares J. Seemann T. Keane J. A.. (2016). SNP-sites: rapid efficient extraction of SNPs from multi-FASTA alignments. Microb. Genom. 2, e000056. doi: 10.1099/mgen.0.000056 28348851 PMC5320690

[B55] Pamilo P. Nei M. (1988). Relationships between gene trees and species trees. Mol. Biol. Evol. 5, 568–583. doi: 10.1093/oxfordjournals.molbev.a040517 3193878

[B56] Pritchard J. K. Stephens M. Donnelly P. (2000). Inference of population structure using multilocus genotype data. Genetics 155, 945–959. doi: 10.1093/genetics/155.2.945 10835412 PMC1461096

[B57] Ronquist F. Teslenko M. van der Mark P. Ayres D. L. Darling A. Höhna S.. (2012). MrBayes 3.2: efficient Bayesian phylogenetic inference and model choice across a large model space. Syst. Biol. 61, 539–542. doi: 10.1093/sysbio/sys029 22357727 PMC3329765

[B58] Rose J. P. Toledo C. A. Lemmon E. M. Lemmon A. R. Sytsma K. J. (2021). Out of sight, out of mind: widespread nuclear and plastid-nuclear discordance in the flowering plant genus *Polemonium* (Polemoniaceae) suggests widespread historical gene flow despite limited nuclear signal. Syst. Biol. 70, 162–180. doi: 10.1093/sysbio/syaa049 32617587

[B59] Simão F. A. Waterhouse R. M. Ioannidis P. Kriventseva E. V. Zdobnov E. M. (2015). BUSCO: Assessing genome assembly and annotation completeness with single-copy orthologs. Bioinformatics 31, 3210–3212. doi: 10.1093/bioinformatics/btv351 26059717

[B60] Smith S. A. Moore M. J. Brown J. W. Yang Y. (2015). Analysis of phylogenomic datasets reveals conflict, concordance, and gene duplications with examples from animals and plants. BMC Evol. Biol. 15, 150. doi: 10.1186/s12862-015-0423-0 26239519 PMC4524127

[B61] Stamatakis A. (2014). RAxML version 8: A tool for phylogenetic analysis and post-analysis of large phylogenies. Bioinformatics 30, 1312–1313. doi: 10.1093/bioinformatics/btu033 24451623 PMC3998144

[B62] Stull G. W. Soltis P. S. Soltis D. E. Gitzendanner M. A. Smith S. A. (2020). Nuclear phylogenomic analyses of asterids conflict with plastome trees and support novel relationships among major lineages. Am. J. Bot. 107, 790–805. doi: 10.1002/ajb2.1468 32406108

[B63] Su C. Duan L. Liu P. Liu J. Chang Z. Wen J. (2021). Chloroplast phylogenomics and character evolution of eastern Asian *Astragalus* (Leguminosae): Tackling the phylogenetic structure of the largest genus of flowering plants in Asia. Mol. Phylogenet. Evol. 156, 107025. doi: 10.1016/j.ympev.2020.107025 33271371

[B64] Sukumaran J. Holder M. T. (2010). DendroPy: A Python library for phylogenetic computing. Bioinformatics 26, 1569–1571. doi: 10.1093/bioinformatics/btq228 20421198

[B65] Tamura M. (1995). “Clematis l,” in Die natürlichen pflanzenfamilien, vol. 17a . Ed. Hiepko P. (Berlin: Duncker und Humbolt), 368–387.

[B66] Thode V. A. Lohmann L. G. Sanmartín I. (2020). Evaluating character partitioning and molecular models in plastid phylogenomics at low taxonomic levels: A case study using *Amphilophium* (Bignonieae, bignoniaceae). J. Syst. Evol. 58, 1071–1089. doi: 10.1111/jse.12579

[B67] Valcárcel V. Wen J. (2019). Chloroplast phylogenomic data support Eocene amphi-pacific early radiation for the Asian palmate core araliaceae. J. Syst. Evol. 57, 547–560. doi: 10.1111/jse.12522

[B68] Vargas O. M. Heuertz M. Smith S. A. Dick C. W. (2019). Target sequence capture in the Brazil nut family (Lecythidaceae): Marker selection and *in silico* capture from genome skimming data. Mol. Phylogenet. Evol. 135, 98–104. doi: 10.1016/j.ympev.2019.02.020 30818022

[B69] Wang W. T. Bartholomew B. (2001). “Clematis l,” in Flora of China, vol. 6 . Eds. Wu C. Y. Raven P. (Beijing: Science Press, St. Louis, Missouri Botanical Garden Press), 333–386.

[B70] Wang W. T. Li L. Q. (2005). A new system of classification of the genus *Clematis* (Ranunculaceae). Acta Phytotax. Sin. 43, 431–488. doi: 10.1360/aps040130

[B71] Wang Y. B. Liu B. B. Nie Z. L. Chen H. F. Chen F. J. Figlar R. B.. (2020). Major clades and a revised classification of *Magnolia* and magnoliaceae based on whole plastid genome sequences *via* genome skimming. J. Syst. Evol. 58, 673–695. doi: 10.1111/jse.12588

[B72] Watson L. E. Siniscalchi C. M. Mandel J. (2020). Phylogenomics of the hyperdiverse daisy tribes: Anthemideae, astereae, calenduleae, gnaphalieae, and senecioneae. J. Syst. Evol. 58, 841–852. doi: 10.1111/jse.12698

[B73] Wen J. Harris A. J. Ickert-Bond S. M. Dikow R. Wurdack K. Zimmer E. A. (2017). Developing integrative systematics in the informatics and genomic era, and calling for a global biodiversity cyberbank. J. Syst. Evol. 55, 308–321. doi: 10.1111/jse.12270

[B74] Wen J. Harris A. J. Kalburgi Y. Zhang N. Xu Y. Zheng W.. (2018). Chloroplast phylogenomics of the new world grape species (*Vitis*, vitaceae). J. Syst. Evol. 56, 297–308. doi: 10.1111/jse.12447

[B75] Wikström N. Bremer B. Rydin C. (2020). Conflicting phylogenetic signals in genomic data of the coffee family (Rubiaceae). J. Syst. Evol. 58, 440–460. doi: 10.1111/jse.12566

[B76] Xiang K. L. Aytaç Z. Liu Y. Espinosa F. Jabbour F. Byng J. W.. (2017). Recircumscription of *Delphinium* subg. *Delphinium* (Ranunculaceae) and implications for its biogeography. Taxon 66, 554–566. doi: 10.12705/663.3

[B77] Xie L. Wen J. Li L. Q. (2011). Phylogenetic analyses of *Clematis* (Ranunculaceae) based on sequences of nuclear ribosomal ITS and three plastid regions. Syst. Bot. 36, 907–921. doi: 10.1600/036364411X604921

[B78] Yang Y. Li Y. Chen Q. Sun Y. Lu Z. (2019). WGDdetector: A pipeline for detecting whole genome duplication events using the genome or transcriptome annotations. BMC Bioinf. 20, 1–6. doi: 10.1186/s12859-019-2670-3 PMC6375192 30760221

[B79] Yang Y. Z. Sun P. C. Lv L. K. Wang D. L. Ru D. F. Li Y.. (2020). Prickly waterlily and rigid hornwort genomes shed light on early angiosperm evolution. Nat. Plants 6, 215–222. doi: 10.1038/s41477-020-0594-6 32094642 PMC8075997

[B80] Yan S. X. Liu H. J. Lin L. L. Liao S. Li J. Y. Pei L. Y.. (2016). Taxonomic status of *Clematis acerifolia* var. *elobata*, based on molecular evidence. Phytotaxa 268, 209–219. doi: 10.11646/phytotaxa.268.3.5

[B81] Yuan T. Wang L. Y. Roh M. S. (2010). Confirmation of *Clematis* hybrids using molecular markers. Sci. Hortic. 125, 136–145. doi: 10.1016/j.scienta.2010.03.005

[B82] Yu X. Q. Yang D. Guo C. Gao L. M. (2018). Plant phylogenomics based on genome-partitioning strategies: Progress and prospects. Plant Divers. 40, 158–164. doi: 10.1016/j.pld.2018.06.005 30740560 PMC6137260

[B83] Zhai W. Duan X. Zhang R. Guo C. Li L. Xu G.. (2019). Chloroplast genomic data provide new and robust insights into the phylogeny and evolution of the ranunculaceae. Mol. Phylogenet. Evol. 135, 12–21. doi: 10.1016/j.ympev.2019.02.024 30826488

[B84] Zhang C. Rabiee M. Sayyari E. Mirarab S. (2018). ASTRAL-III: polynomial time species tree reconstruction from partially resolved gene trees. BMC Bioinf. 19, 15–30. doi: 10.1186/s12859-018-2129-y PMC5998893 29745866

[B85] Zhang R. Wang Y. H. Jin J. J. Stull G. W. Bruneau A. Cardoso D.. (2020). Exploration of plastid phylogenomic conflict yields new insights into the deep relationships of leguminosae. Syst. Biol. 69, 613–622. doi: 10.1093/sysbio/syaa013 32065640 PMC7302050

[B86] Zhang B. W. Xu L. L. Li N. Yan P. C. Jiang X. H. Woeste K. E.. (2019). Phylogenomics reveals an ancient hybrid origin of the Persian walnut. Mol. Biol. Evol. 36, 2451–2461. doi: 10.1093/molbev/msz112 31163451

[B87] Zhao D. N. Ren C. Q. Zhang J. Q. (2021). Can plastome data resolve recent radiations? *Rhodiola* (Crassulaceae) as a case study. Bot. J. Linn. Soc 197, 513–526. doi: 10.1093/botlinnean/boab035

[B88] Zimmer E. A. Wen J. (2015). Using nuclear gene data for plant phylogenetics: Progress and prospects II. next-gen approaches. J. Syst. Evol. 53, 371–379. doi: 10.1111/jse.12174

[B89] Zou X. H. Ge S. (2008). Conflicting gene trees and phylogenomics. J. Syst. Evol. 46, 795–807. doi: 10.3724/SP.J.1002.2008.08081

