# Supplementary figures and images for: Genome-partitioning strategy, plastid and nuclear phylogenomic discordance, and its evolutionary implications of Clematis (Ranunculaceae)
Source: Front Plant Sci. 2022 Nov 14;13:1059379. doi: 10.3389/fpls.2022.1059379 (PMC9703796; doi:10.3389/fpls.2022.1059379)

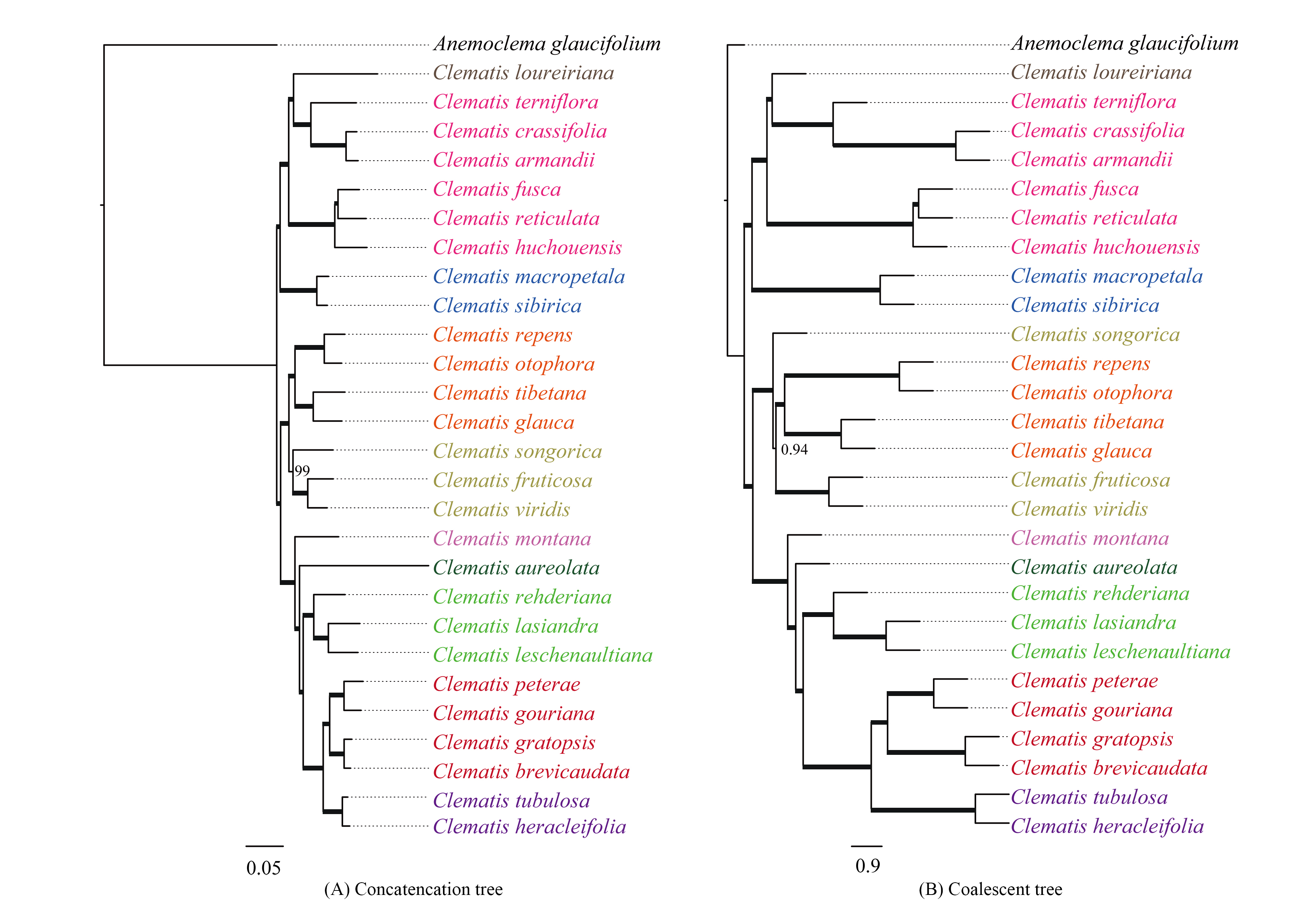

Supplement: Supplementary Figure 1 — Phylogenetic trees constructed by concatenated and coalescence-based methods based on SCOG3000 dataset. [file Image_1.jpeg]

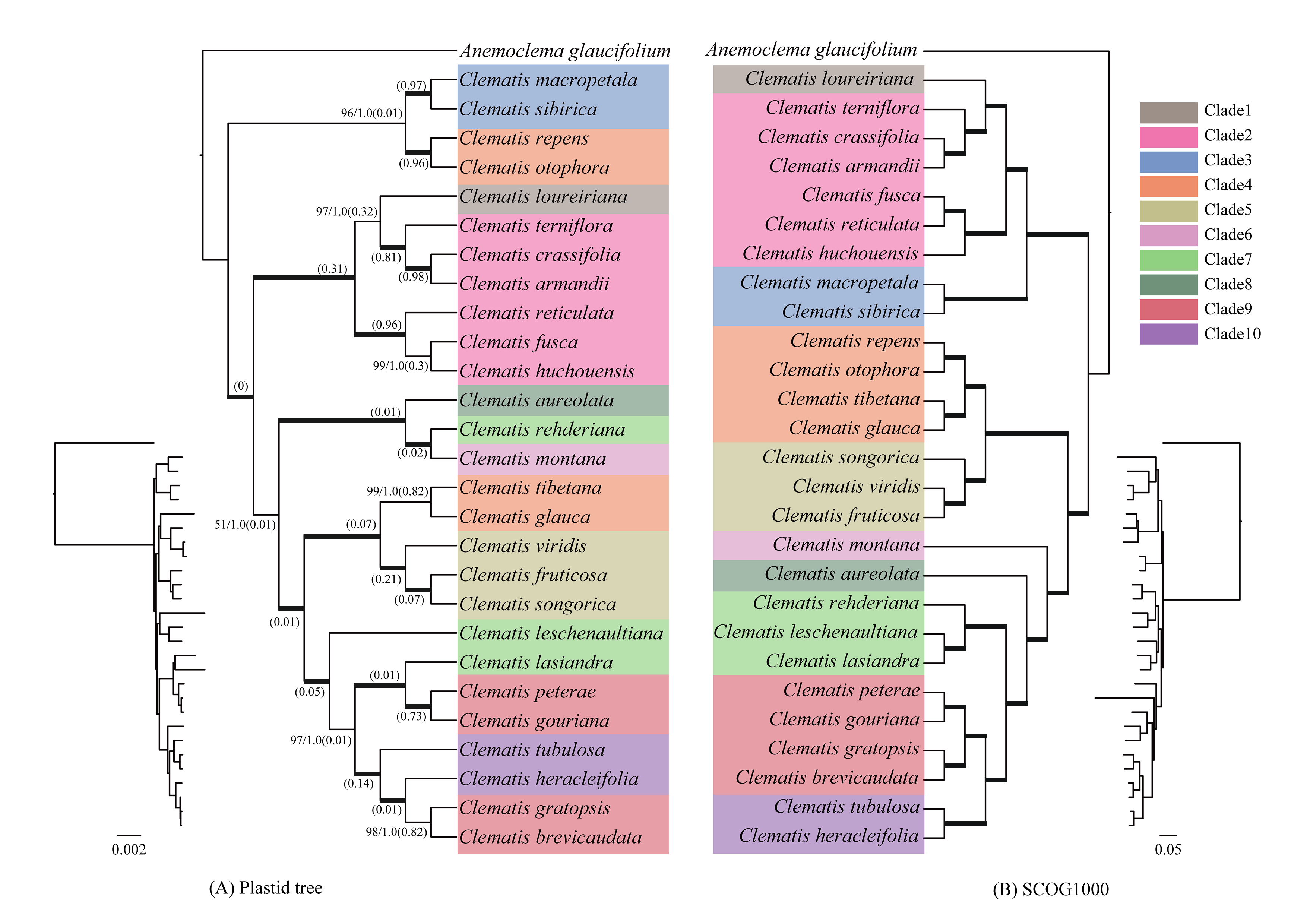

Supplement: Supplementary Figure 2 — Bayesian phylogeny (A) of Clematis inferred from the plastid genome data and maximum likelihood phylogeny (B) inferred from of SCOG1000 data. Cyto-nuclear discordance is shown. [file Image_2.jpeg]

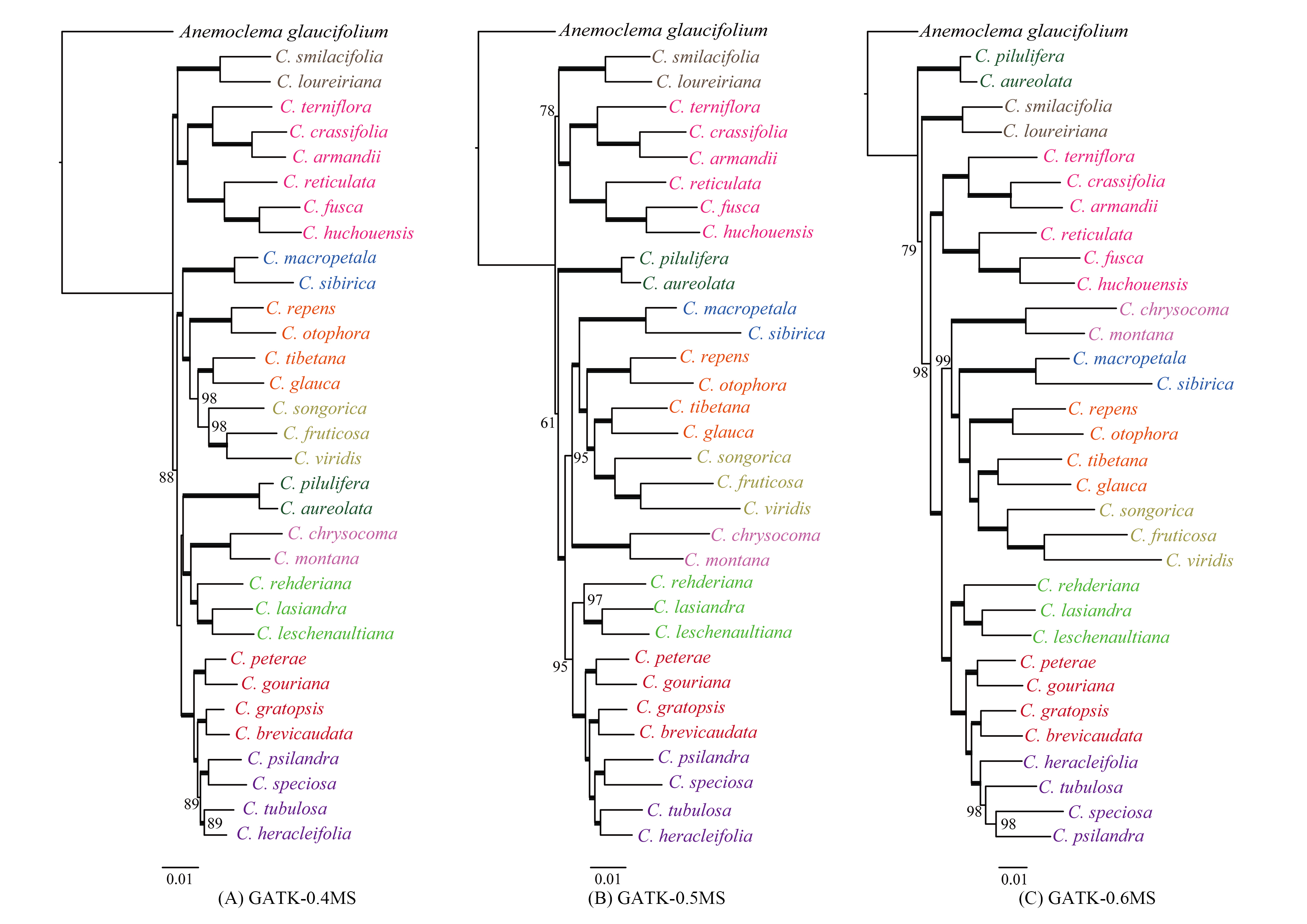

Supplement: Supplementary Figure 3 — Maximum likelihood phylogenetic trees constructed from three nuclear SNPs data matrices obtained by GATK pipeline. [file Image_3.jpeg]

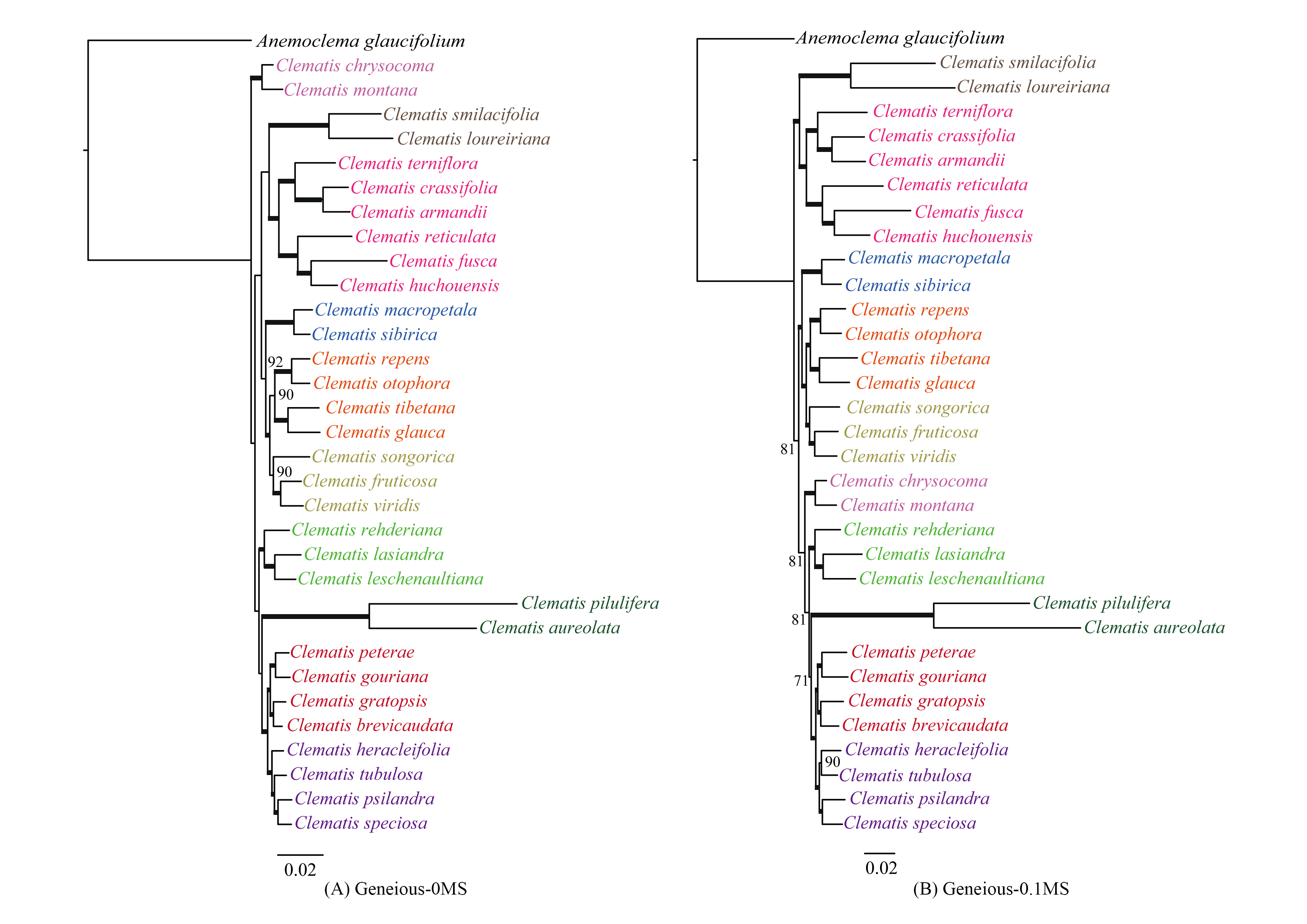

Supplement: Supplementary Figure 4 — Maximum likelihood phylogenetic trees constructed from two nuclear SNPs data matrices obtained by Geneious pipeline. [file Image_4.jpeg]
